# Supplementary material for: Hepatitis C Virus NS5A Activates Mitophagy Through Cargo Receptor and Phagophore Formation
Source: Pathogens. 2024 Dec 23;13(12):1139. doi: 10.3390/pathogens13121139 (PMC11680023; doi:10.3390/pathogens13121139)
Supplement: Supplementary file 1 [file pathogens-13-01139-s001.zip › Supplemental Figures.pdf]

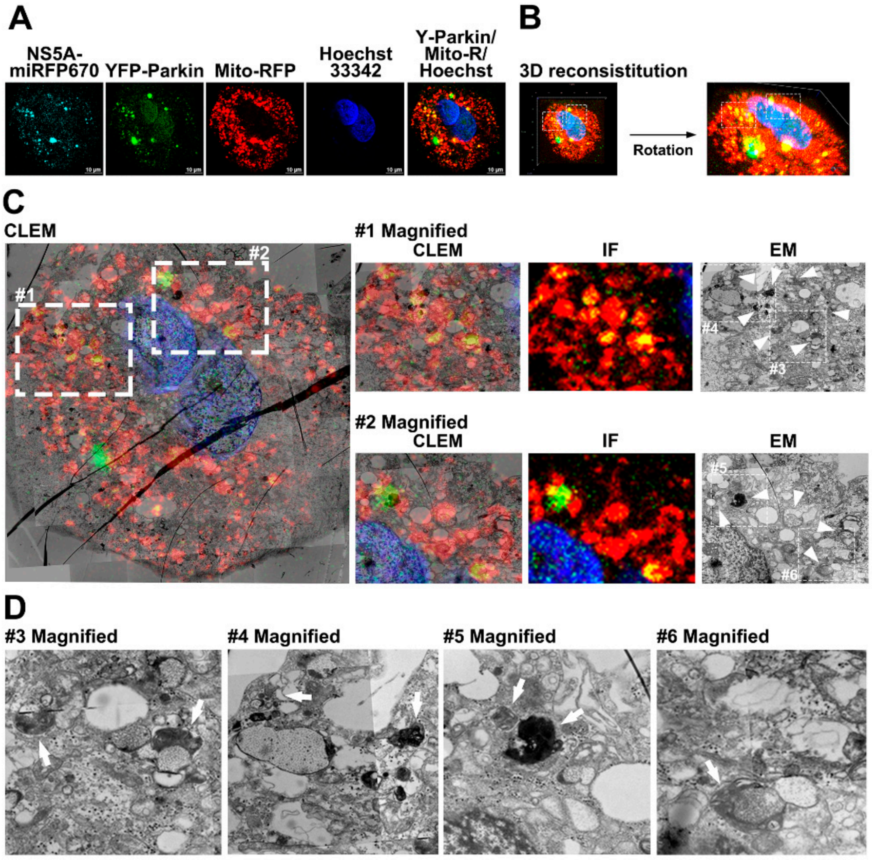

Supplemental Figure S1. CLEM analysis of the HCV NS5A-induced mitochondrial translocation of Parkin: (A) Huh7/RFP-Parkin/Mito-GFP cells were transduced with lentivirus expressing HCV NS5A-miRFP670. Forty-eight hours later, the cells were analyzed via confocal microscopy. (B) The Z-stacks of the confocal micrograph shown in (A) were assembled and deconvoluted into a 3D image. The white dashed boxes indicate the Mito-GFP-expressing mitochondria with RFP-Parkin translocation. (C) The aligned IF and CLEM image of cells from (B) is presented. The white dashed boxes in the left panel are enlarged and shown in the magnified images in the right panel. The white arrowheads indicate the degradative mitochondria in which Parkin translocates. (D) The enlarged images show the magnified white dashed boxes in the EM of (C). The white arrows indicate the phagophores wrapped around deformed mitochondria.

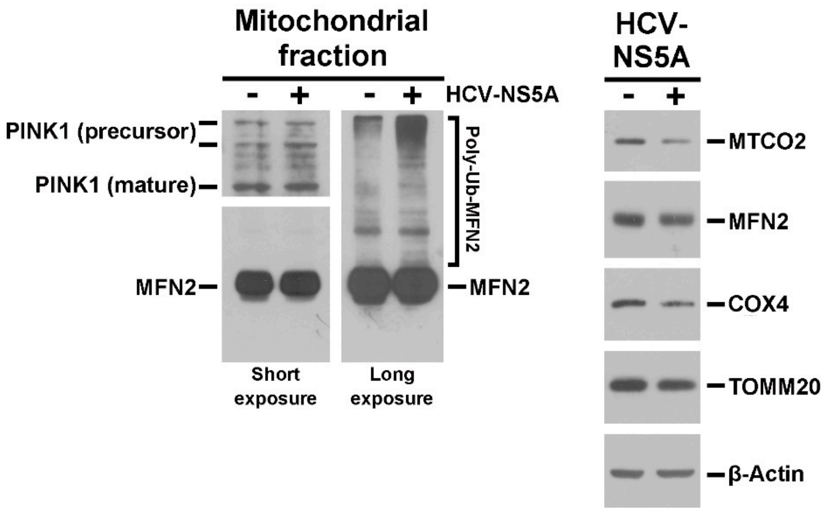

Supplemental Figure S2. PINK1 stabilization and MFN2 ubiquitination on mitochondria in HCV NS5A-expressing cells: Huh7 cells were transduced with lentivirus expressing HCV NS5A-mTagBFP2 for forty-eight hours. The cytosolic and mitochondrial fractions of the cells were biochemically isolated using a mitochondria isolation kit (89874, Thermo Fisher Scientific). The cytosolic and mitochondrial fractions were analyzed for protein expression via SDS–PAGE and western blot analysis.

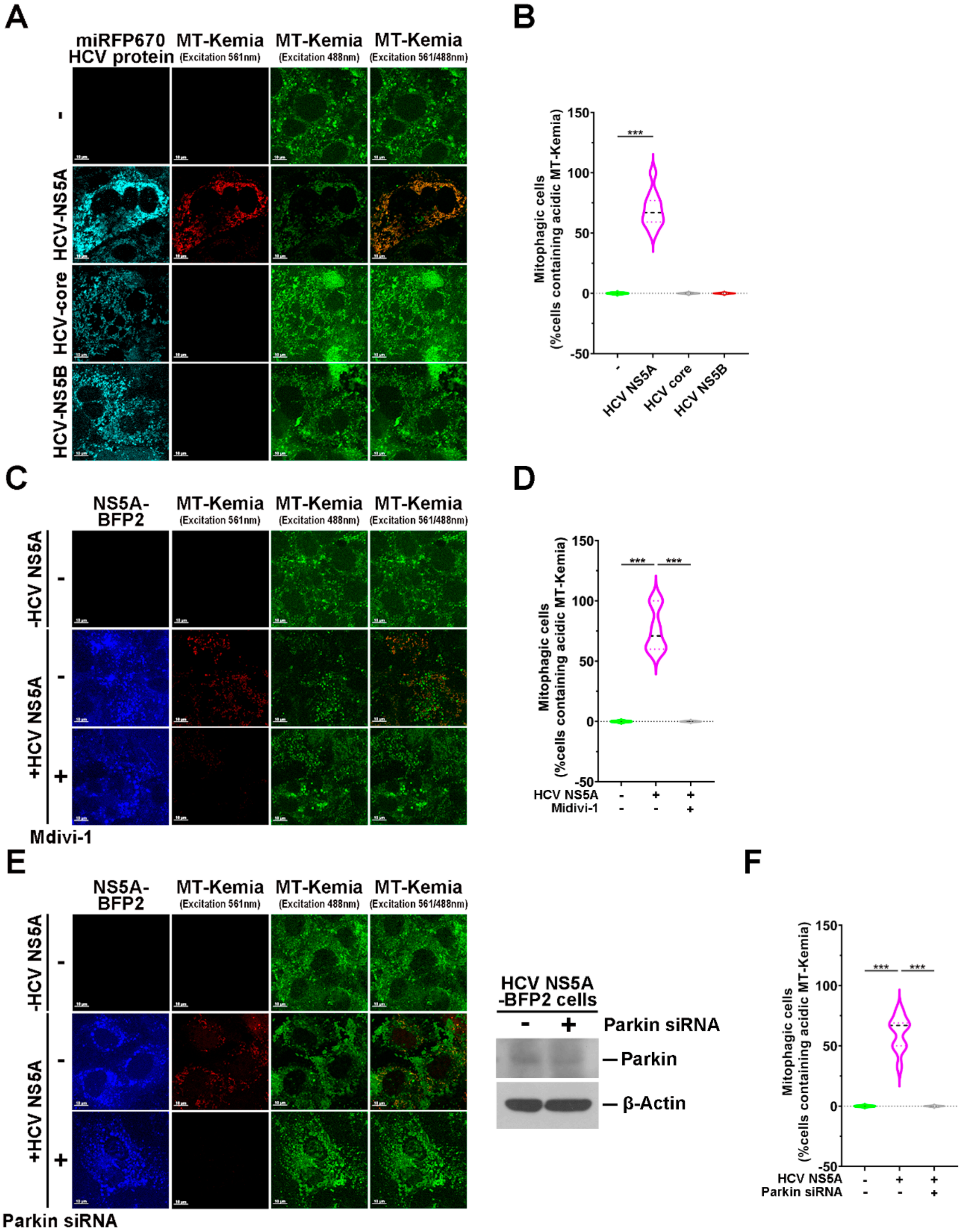

Supplemental Figure S3. Specific induction of mitophagic degradation by HCV NS5A: (A-B) A: Huh7/MT-Keima cells were transduced without (-) or with (+) pTRIP-HCV NS5A-miRFP670, pTRIP-miRFP670-HCV core, and pTRIP-miRFP670-HCV NS5B lentiviruses. Forty-eight hours later, the cells were analyzed via confocal microscopy at short (488 nm) and long (561 nm) excitation wavelengths. (B) The percentage of cells containing acidic MT-Keima (excitation at 561 nm) was quantified. (C-D) Huh7/MT-Keima cells were transduced with (+) or without (-) pTRIP-HCV NS5A-mTagBFP2 for forty hours. Then, the HCV NS5A-mTagBFP2-transduced MT-Keima cells were treated without (-) or with (+) Mdivi-1 at a concentration of 10 μM. Eight hours later, the cells were analyzed and quantified for fluorescence of MT-Keima as described above. (E-F) Huh7/MT-Keima cells were transduced with (+) or without (-) pTRIP-HCV NS5A-mTagBFP2 for twelve hours. Then, the HCV NS5A-mTagBFP2-transduced MT-Keima cells were transfected without (-) or with (+) Parkin siRNA duplexes by electroporation with the Neon MicroPorator MP-100. Thrity-six hours later, the cells were analyzed and quantified for fluorescence of MT-Keima as described in (A-B). The protein expressions of Parkin and β-actin were analyzed via SDS–PAGE and western blot analysis. The data shown in (B), (D), and (F) represent the mean ± SEM (n=10, ***P<0.001).

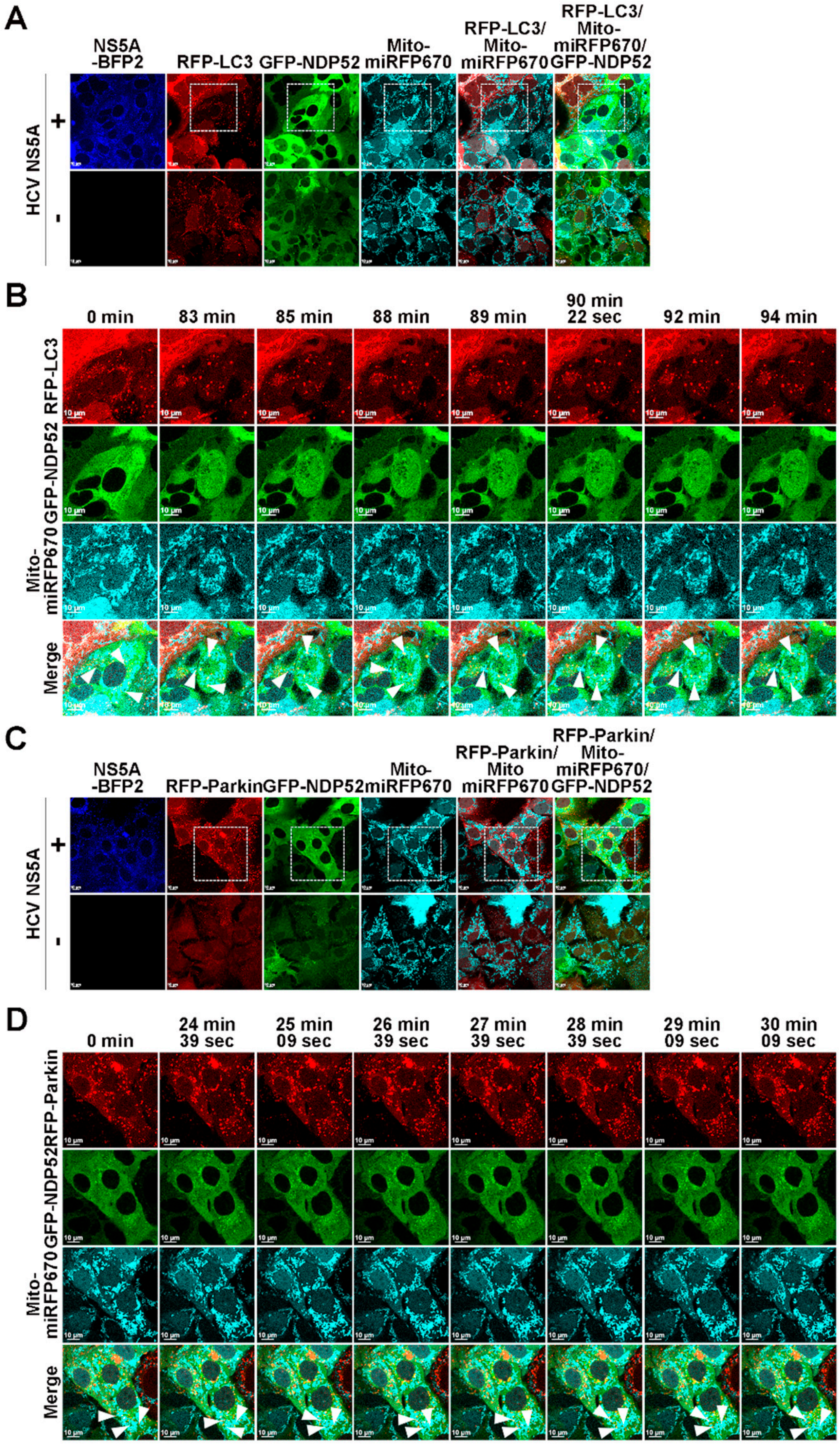

Supplemental Figure S4. Time-lapse live-cell imaging of NDP52 recruitment into HCV NS5A-activated mitophagy: (A-B) A: Huh7/RFP-LC3/Mito-miRFP670/GFP-NDP52 cells were established as described in Figure 6A. Then, the cells were transduced with (+) or without (-) pTRIP-HCV NS5A-mTagBFP2 lentivirus for forty-eight hours and analyzed via confocal microscopy. B: The selected live imaging frames show the magnified area in the white dashed box of the upper panel in (A). The white arrowheads indicate the recruitment of GFP-NDP52 to Mito-miRFP670-labeled mitochondria before sequestration by RFP-LC3 puncta. (C-D) C: Huh7/RFP-Parkin/Mito-miRFP670/GFP-NDP52 cells were established as described in Figure 6E. Then, the cells were transduced with (+) or without (-) pTRIP-HCV NS5A-mTagBFP2 lentivirus for forty-eight hours and analyzed via confocal microscopy. D: The selected live imaging frames show the magnified area in the white dashed box of the upper panel in (C). The white arrowheads indicate the recruitment of GFP-NDP52 to Mito-miRFP670-labeled mitochondria after RFP-Parkin translocation.

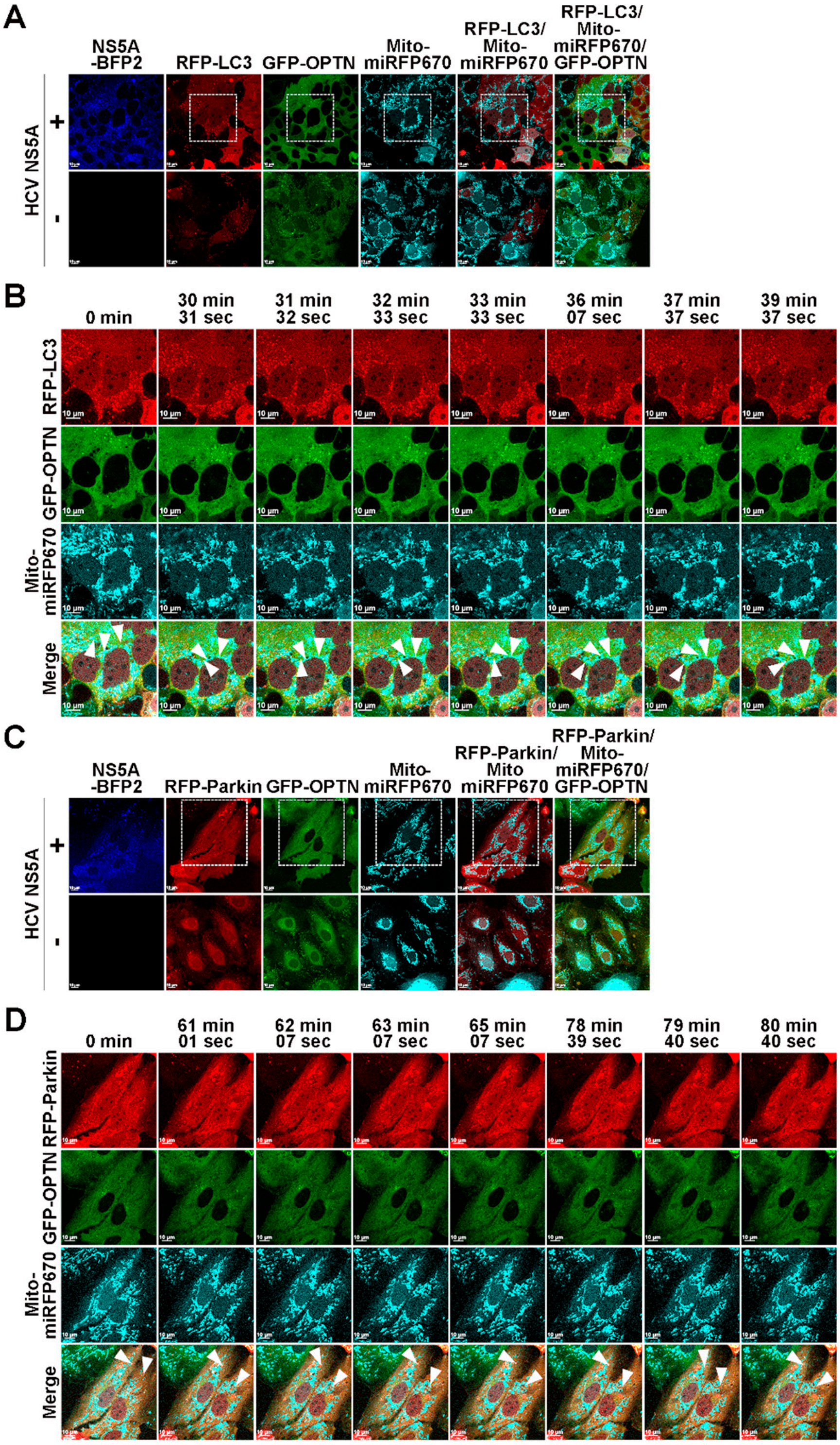

Supplemental Figure S5. Time-lapse live-cell imaging of OPTN recruitment into HCV NS5A-activated mitophagy: (A-B) A: Huh7/RFP-LC3/Mito-miRFP670/GFP-OPTN cells were established as described in Figure 6C. Then, the cells were transduced with (+) or without (-) pTRIP-HCV NS5A-mTagBFP2 lentivirus for forty-eight hours and analyzed via confocal microscopy. B: The selected live imaging frames show the magnified area in the white dashed box of the upper panel in (A). The white arrowheads indicate the recruitment of GFP-OPTN to Mito-miRFP670-labeled mitochondria before sequestration by RFP-LC3 puncta. (C-D) C: Huh7/RFP-Parkin/Mito-miRFP670/GFP-OPTN cells were established as described in Figure 6G. Then, the cells were transduced with (+) or without (-) pTRIP-HCV NS5A-mTagBFP2 lentivirus for forty-eight hours and analyzed via confocal microscopy. D: The selected live imaging frames show the magnified area in the white dashed box of the upper panel in (C). The white arrowheads indicate the recruitment of GFP-OPTN to Mito-miRFP670-labeled mitochondria after RFP-Parkin translocation.

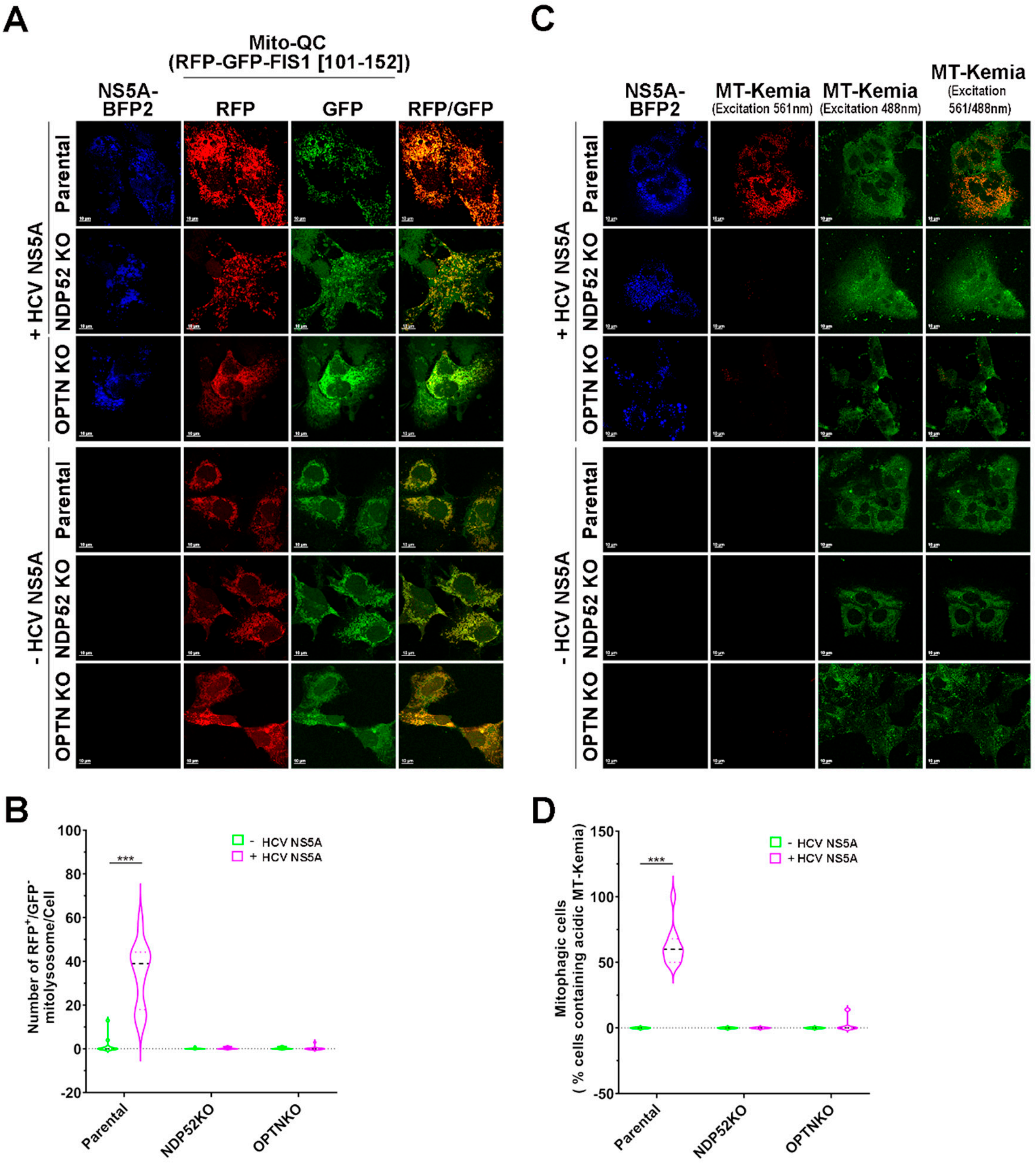

Supplemental Figure S6. Requirement of NDP52 and OPTN for HCV NS5A-activated mitophagy: (A-B) A: Huh7/NDP52KO, Huh7/OPTNKO, and Huh7/Parental cells were established as described in the "Materials and Methods" section. These cells were transduced with pTRIP-Mito-QC lentivirus and then transduced with (+) or without (-) pTRIP-HCV NS5A-mTagBFP2 lentivirus. Forty-eight hours later, cells were analyzed via confocal microscopy. B: The number of RFP^+/GFP^- mitolysosomes in each sample was quantified. The data are presented as means ± SEMs (n=10, ***P<0.001). (C-D) C: Huh7/NDP52KO, Huh7/OPTNKO, and Huh7/Parental cells were transduced with pTRIP-MT-Keima lentivirus and then transduced with (+) or without (-) pTRIP-HCV NS5A-mTagBFP2 lentivirus. Forty-eight hours later, cells were analyzed via confocal microscopy at short (488 nm) and long (561 nm) excitation wavelengths. D: The percentage of cells containing acidic MT-Keima (excitation at 561 nm) in each sample was quantified. The data are presented as means ± SEMs (n=10, ***P<0.001).
